# Causes of Death and Survival in Alcoholic Cirrhosis Patients Undergoing Liver Transplantation: Influence of the Patient’s Clinical Variables and Transplant Outcome Complications

**DOI:** 10.3390/diagnostics11060968

**Published:** 2021-05-27

**Authors:** J. M. Bolarín, M. D. Pérez-Cárceles, J. P. Hernández del Rincón, A. Luna, A. Minguela, M. Muro, I. Legaz

**Affiliations:** 1 Department of Legal and Forensic Medicine, Faculty of Medicine, Biomedical Research Institute (IMIB), Regional Campus of International Excellence “Campus Mare Nostrum”, University of Murcia, 30100 Murcia, Spain; bolarin5@hotmail.com (J.M.B.); mdperez@um.es (M.D.P.-C.); jphrincon@um.es (J.P.H.d.R.); aurluna@um.es (A.L.); 2 Forensic Pathology Department, Institute of Legal and Forensic Medicine, 30100 Murcia, Spain; 3 Centro de Investigación Biomédica en Red de Enfermedades Hepáticas y Digestivas (CIBERehd), Immunology Service, Instituto Murciano de Investigación Biosanitaria (IMIB), Hospital Clínico Universitario Virgen de la Arrixaca (HCUVA), 30120 Murcia, Spain; alfredo.minguela@carm.es (A.M.); manuel.muro@carm.es (M.M.)

**Keywords:** alcoholic cirrhosis, causes of death, forensic medicine, human clinical toxicology, liver transplant, medico-legal autopsy, survival

## Abstract

Background. Clinical and molecular mechanisms involved in the cause and time of death of alcoholic cirrhosis (AC) patients undergoing liver transplantation (LT) are not entirely understood. In sudden death cases, judicial autopsy practice is mandatory for determining the cause and circumstances of death. The medico-legal autopsy data are essential for helping health authorities to guide future public health activities, assess the effectiveness of health systems, and adopt the necessary preventive measures to improve and adapt the treatments in order to increase these patients’ survival. Objective. Our study aimed to determine the different clinical and sociodemographic causes that influence the different causes of death and the short- and long-term survival of AC patients undergoing liver transplantation. Methods. A total of 122 deceased AC patients undergoing LT were analyzed at different times post-transplantation. The main pre- and post-transplant complications were analyzed in relation to the cause of death and the patient’s survival, as well as the causes and time at which the patient’s death occurred. Results. A total of 53.3% of non-sudden death was observed. A large number of the deaths of AC patients undergoing transplantation were due to non-sudden death, sepsis, and graft failure (GF), the main causes of death in the sample being similar in both sexes. In non-sudden deaths, there were no significant differences between the death rates either related or not related to the liver transplant. Sepsis was the main cause, with the highest percentage (21.3%) of mortality, followed by GF (18.9%) and multiorgan failure (15.6%) at ten years. Furthermore, our results showed how pre-transplant clinical complications, such as viral infections and encephalopathy, influence the age at which multiorgan failure occurs in the transplanted patient. Conclusion. Multiorgan failure is the leading cause of sudden death, with higher mortality during the first year after transplantation, followed by sepsis and GF. Our results show the vulnerability of AC patients, both in the hospital period after the transplant and outside.

## 1. Introduction

Clinical and molecular mechanisms involved in the cause of death and survival of AC patients are not entirely understood, but it is generally believed that many factors can influence these patients’ deaths [1,2,3,4,5]. AC can induce cardiac arrhythmias [6,7,8,9,10] and ventricular tachycardia [11,12,13], increase the risk for infection with hepatitis C [14], and increase the risk of different diseases [15,16].

On the other hand, the cause of death and survival of AC patients undergoing liver transplantation could be influenced by different pre-transplant and post-transplant complications. [1,2]. The survival rate of transplanted AC patients as a primary disease has increased dramatically in recent years, rendering this liver disease one of the best indications for liver transplantation (LT) [2,17,18]. Nevertheless, patients should undergo careful post-transplant risk assessment for rejection development; viral recurrence; alcoholic relapse; and, above all, extreme precautions to prevent the patient ultimately from dying [19].

Various studies have attempted to analyze the causes of death of AC patients undergoing liver transplantation [14,20]. Causes of alcohol-related sudden death include intracranial bleeds, heart blocks, metabolic acidosis with a cardiac standstill, and exsanguinating gastrointestinal bleed [21,22,23]. In sudden death cases, judicial autopsy practice is mandatory to determine the cause and circumstances of the death [19,24,25].

The medico-legal autopsy data are essential in epidemiological surveys [26,27], as they help health authorities to guide future public health activities and to assess the effectiveness of health systems, as well as to adopt the necessary preventive measures to improve and adapt the treatments with the purpose of increasing the survival of these patients [28].

However, despite these relatively sporadic scientific publications, the frequent causes of death due to alcoholic disease in liver transplant patients are not exact, and measures to prevent unexpected death in AC patients after LT have not yet been established due to a lack of large scale studies concerning such cases.

Our study aimed to determine the different clinical and/or sociodemographic causes that influence the different causes of death and the short- and long-term survival of AC patients undergoing liver transplantation. Their knowledge is vital for adopting the necessary preventive measures to establish a healthcare routine screening to improve and adapt the treatments to increase their survival.

## 2. Material and Methods

### 2.1. Patient Enrollment

**Figure 1:**
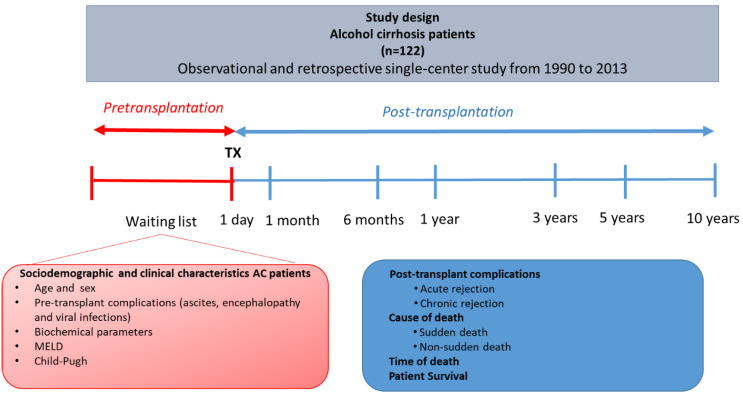
Scheme of the study design and the variables analyzed in each phase of the study. N, number of patients; Tx, transplantation MELD, model for end-stage liver disease.

A cohort of 122 consecutive Caucasian AC patients undergoing LT at the University Clinic Hospital ‘Virgen de la Arrixaca’ in the Murcia Region (Spain) from 1990 to 2013 was retrospectively reviewed. In this observational and retrospective single-center study. The median age was 53.1 ± 8.9 (mean years ± SD) in this cohort. Figure 1 shows a diagram of the design and the different variables considered in this study.

The sociodemographic and clinical characteristics of the cohort analyzed in this study are shown in Table 1. For this study, the AC patients were classified by sex, viral infection, cause, and time of death.

The inclusion criteria were primary LT without any prior history of other organ transplants, ABO compatibility, HIV-negativity, and whole liver allograft. Our AC patients’ leading causes of death until ten years post-transplant were studied and classified by sex.

All patients provided informed consent after being given information on the follow-up data. The institutional ethical committee approved the study’s protocol in accordance with the principles of the Declaration of Helsinki. The research was carried out in compliance with the Helsinki Agreement, and the protocol was approved by the HCUVA Ethics Committee (PI15/01370). Current regulations that guarantee the confidentiality of personal data and its automated treatment were respected.

### 2.2. Diagnostic Criteria of Alcoholic Cirrhosis

AC was diagnosed using clinical, radiologic, and biochemical parameters [29]. AC patients had a history of consuming alcohol above the accepted safe limits before the diagnosis of cirrhosis. In the case of the low self-reporting of alcohol intake, family statements were considered. In other cases, the disease remained undetected until the second stage of decompensated cirrhosis, when complications such as ascites, upper gastrointestinal bleeding, and encephalopathy appeared. Cases of presumed cirrhosis were verified using advanced imaging and measurement methods. The degree of hepatic fibrosis of all patients included in this study was grade F4 (METAVIR score) at the time of inclusion on the waitlist for a liver transplant.

### 2.3. Main Causes and Time of Death in Alcoholic Cirrhosis Patients

The main causes and time of death of AC patients were determined by medical death certificates or medico-legal autopsy until ten years after LT. AC patients with liver transplants are subject to monitoring and control throughout their lives. In these patients, the autopsy and analysis of liver explants is standard practice. In the case of unknown deaths, the autopsy was not performed.

Causes of death were the primary reason for death listed by the physician involved in the patient’s care. The main causes of death were classified into three main groups in this study: sudden death, non-sudden death, and unknown death (no records of the type or cause of death were found). Sudden death was defined as death, often instantaneous, within 24 h of symptom onset, when information from hospital records, forensic medical reports, or general practice allowed this definition to be made with certainty. Non-sudden death was recorded when there was clear evidence that death had occurred >24 h after the onset of symptoms [30].

On the other hand, to ascertain the possible influence of the transplanted liver on the cause of death, death causes were also grouped for analysis into two groups: liver-related and non-liver-related. On the other hand, causes of death were analyzed at different times after LT: ≤1 month, >1–6 months, and ≥6 months–10 years.

### 2.4. Viral Infection, Ascites, and Hepatic Encephalopathy Diagnosis

Pre-infection with hepatitis C virus (HCV) was determined using a qualitative immunoassay (AxSYM HCV v3.0; Abbott, Wiesbaden Delkenheim, Germany) to detect anti-HCV antibodies. The findings were verified by immunoblotting (recombinant immunoblot assay) or reverse transcription and polymerase chain reaction (PCR) (REAL; Durviz, Valencia, Spain) as indicated by the manufacturer. Hepatitis B viral infection (HBV) was determined using a radioimmunological approach to measure the HBV surface antigen (SorinBiomedica, Perugia, Italy). According to the presence or absence of viral infections, AC patients were classified into viral (*n* = 33) and non-viral AC patient groups (*n* = 89). Ascites and alcoholic encephalopathy were also diagnosed according to previous studies [31,32].

### 2.5. Ascites and Hepatic Encephalopathy Diagnosis

Ascites was diagnosed, establishing three different grades ranging from low (grade I) to high involvement [33]. Alcoholic encephalopathy was diagnosed and classified into four different grades ranging from low (grade I) to high (grade IV) [32]. In some patients, it was not possible to know the degree of ascites or encephalopathy.

### 2.6. Statistical Analyses

Demographic data and findings were stored in the database (Microsoft Access 2.0; Microsoft Corporation, Seattle, WA, USA), with analyses performed using SPSS version 20.0 software (SPSS Software Inc., Chicago, IL, USA). Both findings are expressed as ± SD or as a percentage. Pearson’s chi-square and 2-tailed Fisher’s exact tests were used to compare categorized variables between the groups. Kaplan–Meier and log-rank were used to compare short- and long-term AC survival discrepancies between 1 month and 3 years. A level of *p* < 0.05 was accepted as statistically significant. Odds ratios (OR) and their 95% confidence intervals (CI) were calculated to estimate relative risk. Logistic regression multivariate analysis and multivariate survival analysis using Cox regression methods were used to examine the factors associated with different causes of deaths and survivals.

## 3. Results

### 3.1. Sociodemographic and Clinical Characteristics

The socio-demographic and main clinical characteristics (*n* = 122) of the total cohort of deceased AC patients are shown in Table 1. The mean age of the total cohort immediately before liver transplant was 53.1 ± 8.9 (mean years ± SD).

The main pre- and post-transplant complications were also studied in established patient groups. Ascites (82%) was the complication with the highest frequency found in the total cohort, followed by encephalitis (40.4%) and viral infections (27%).

Regarding the mean ages of these groups of patients, it is worth highlighting statistically significant differences (*p* < 0.0001) between the mean ages of the patients with encephalopathy who had a higher mean age (56.7 ± 2.0; mean ± SD), followed by the patients with ascites (54.2 ± 1.3; mean ± SD), and finally with viral infection (51.3 ± 1.9; mean ± SD). Similar observations were found in the groups of male AC patients (*p* < 0.0001) and the group of patients without viruses (*p* < 0.0001). On the contrary, in AC patients with viral infections (*n* = 33), the mean age at which they also presented with ascites (50.4 ± 2.0; mean ± SD) was higher than the mean age at which they presented with encephalopathy (47.2 ± 2.8; mean ± SD), this difference reached statistical significance (*p* = 0.012).

The mean age of patients with ascites and encephalopathy and without viruses was higher than those without viral infection (*p* = 0.024 and *p* = 0.002, respectively). The main causes of death classified in sudden, non-sudden, or unknown causes were analyzed by sex and presence of pre-transplant virus (HCV and HCB) at ten years after LT (Table 2).

The mean ages of the AC patients were not also did not show significant differences by sex (*p* = 0.274), although there was statistical difference with presence or absence of viruses (*p* = 0.019), being the mean age of the virus-free patients older.

In third place, causes of death were also analyzed, observing that 53.3% of registered deaths corresponded to non-sudden deaths (53.3%), followed by 27.9% of sudden deaths, and other unknown causes (18.9%). No statistically significant differences were observed in these groups according to the sex or age of the patients (*p* = 0.080 and *p* = 0.104, respectively). Despite the analysis in virus-infected and non-infected patients, the patient’s mean age with sudden-death was higher than the rest of the causes of death in all cases (*p* < 0.0001).

Likewise, the time of death at different post-transplant periods was also analyzed. An increase in post-transplant deaths was observed from the beginning of LT (≤1 month) to ˃1–6 months post-transplant (37% of deaths), reaching up to 51.3% in the period of ≥6 months to 10 years, this difference being statistically significant (*p* = 0.049). This same trend was also observed depending on sex (*p* = 0.044 and 0.013, respectively). The analysis of the presence of viruses showed an increase in the mean age of the virus-free patients who died between 1-6 months compared to those who presented with viral infection in the same period of death (*p* = 0.003).

### 3.2. Different Causes of Death of AC Patients at the Short and Long Term

**Figure 2:**
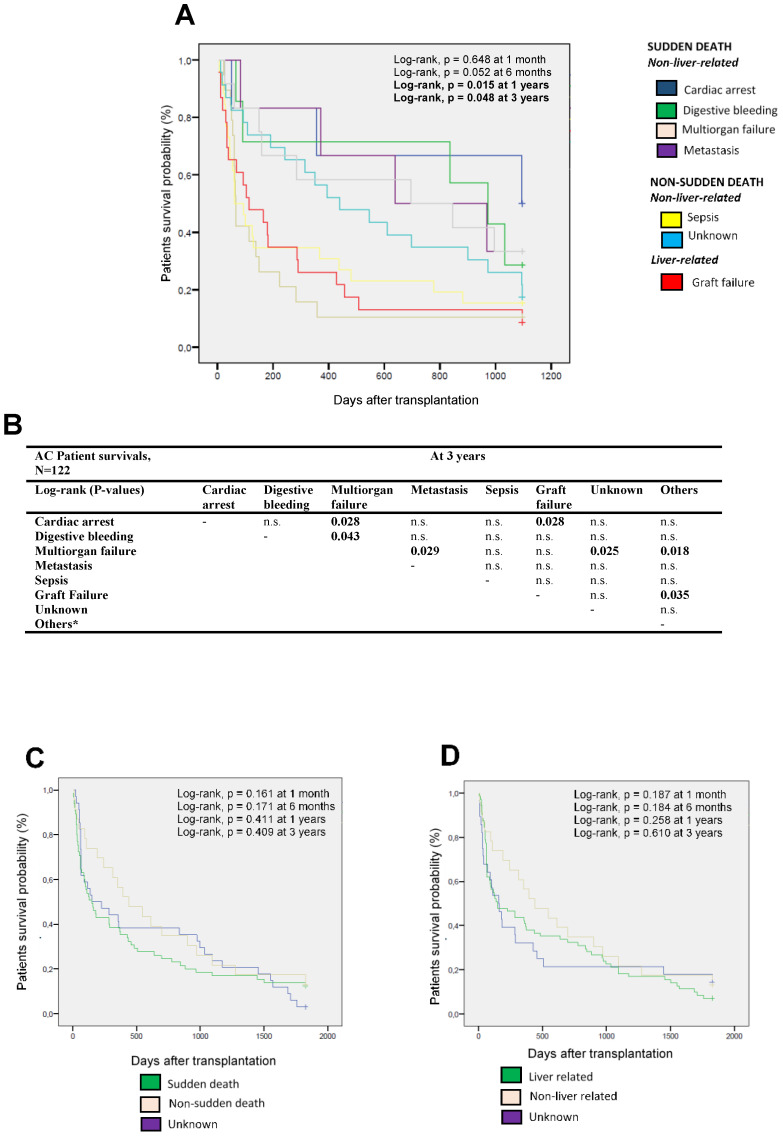
Kaplan–Meier patient survival curves according to the main cause of deaths in AC patients. (**A**) Kaplan–Meier patient survival curves according to the main cause of death in the total AC patients for LT in the short- and long-term over three years. (**B**) Comparative table showing the statistically significant differences between the different causes of death at three years. (**C**) Kaplan–Meier patient survival curves comparing sudden and non-sudden death. (**D**) Kaplan–Meier patient survival curves comparing liver- and non-liver-related death. AC, alcoholic cirrhosis; n.s. no statistical significance. * Others include: lung edema, pharynx neoplasia, pancreatitis, pneumonia, chronic rejection, primary dysfunction, and HCV relapse. HCV, hepatitis C virus; HBV, hepatitis B virus.

The main causes of death of the AC patients at different times after LT were analyzed (Table 3, Figure 2). The causes of death were grouped according to sudden death or non-sudden death and according to liver or non-liver related.

Our results showed a percentage of 28.5% of sudden deaths not related to the liver at one year post-transplant. Multiorgan failure was the leading cause (81.0%). Non-sudden deaths were the second cause with 55.4%, of which 28.4% were not related to the liver, highlighting sepsis (81%). On the other hand, 27% were deaths related to liver, highlighting GF (90%). Finally, 16.2% of the deaths were due to unknown causes. Similar trends in the causes of death were observed at all points of time analyzed in this study. At ten years post-transplantation, sepsis (26/122; 21.3%) was observed as the main cause of non-liver related, non-sudden death. On the other hand, in the analysis of non-sudden death, the most frequent cause related to the liver was multiorgan failure (23/122; 15.6%), and within the causes related to the liver, the most frequent cause was GF (23/122; 18.9%). No statistical differences were found between both sexes.

Subsequently, the different survival of the AC patients was compared according to the different causes of death (Figure 2A), only observing statistically significant differences at 1 and 3 years post-transplantation (log-rank, *p* = 0.015 and *p* = 0.048, respectively).

The causes of death of the patients were compared at three years after transplantation, observing how the cause of death as multiorgan failure differed significantly from cardiac arrest (*p* = 0.028), digestive bleeding (*p* = 0.043), metastasis (*p* = 0.029), unknown cause (*p* = 0.025), and other causes of death (*p* = 0.018) (Figure 2B). Patient survival as a function of sudden and non-sudden death was also analyzed (Figure 2C), not observing any statistically significant differences at the different points of time analyzed. Similar observations were obtained when comparing mortality according to liver or non-liver related causes of death (Figure 2D).

**Figure 3:**
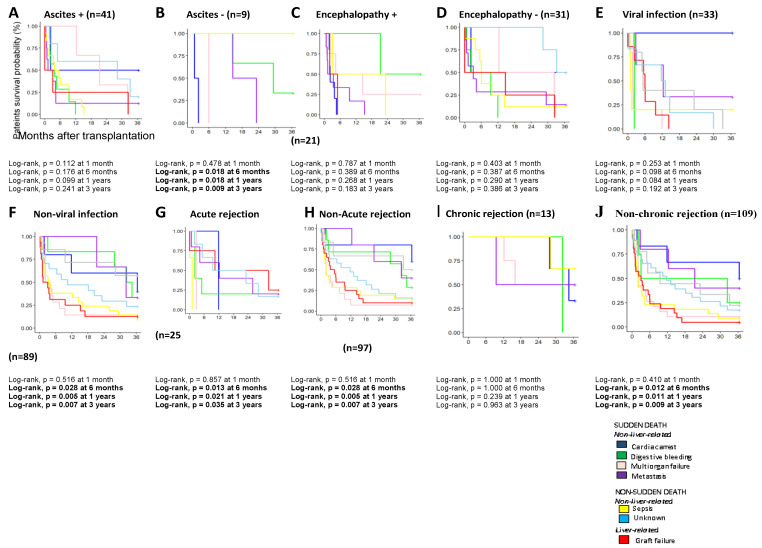
Kaplan–Meier patient survival curves according to the main cause of death in AC patients with and without viral infections (VHC or VHB) in the short- and long-term. (**A**,**B**) Kaplan–Meier patient survival curves comparing AC patients with HCV and without HCV. (**C**,**D**) Kaplan–Meier patient survival curves comparing AC patients with and without HBV. (**E**,**F**) Kaplan–Meier patient survival curves comparing AC patients with and without viral infections (HCV plus HBV). AC, alcoholic cirrhosis; HCV, hepatitis C virus; HBV, hepatitis B virus. (**G**,**H**) Kaplan–Meier patient survival curves comparing AC patients with and without acute rejection. (**I**,**J**) Kaplan–Meier patient survival curves comparing AC patients with and without chronic rejection.

Finally, the influence of ascites, encephalopathy, viral infections, acute or chronic rejection in the AC patient, and the different causes of death were also analyzed (Figure 3).

The presence of pre-transplant complications (ascites or encephalopathy), pre-transplantation viral infections, and the presence of graft rejection do not seem to significantly influence survival according to the different causes of death in the short- or long-term (Figure 3A,C,E,G,I).

On the other hand, AC patients without ascites showed early death from 6 months to 3 years of cardiac arrest and multiorgan failure (log-rank, *p* = 0.018 and 0.009; Figure 3B). However, patients who did not present encephalopathy did not show significant differences in survival according to the cause of death (Figure 3C).

AC patients with viral infection showed (Figure 3E) early deaths in the first months after transplantation due to digestive bleeding, multiorgan failure, and sepsis. Patients without viral infection showed similar causes of death to the total cohort previously analyzed (sepsis, multiorgan failure, and GF), obtaining statistically significant differences between the different causes of death from 6 months t until 3 years post-transplant (log-rank, *p* = 0.012, and *p* = 0.022, respectively; Figure 3F).

Regarding the patients who underwent acute rejection, an increase in mortality in the first months after transplantation and differences between the diverse causes of death from 6 months after transplantation were observed (Figure 3G). On the contrary, in the patients who did not undergo acute rejection, a more prolonged patient survival was observed regardless of the cause of death (Figure 3H). Similar observations were found when the patient presented chronic rejection (Figure 3J). This fact was obtained by multivariate logistic Cox regression (*p* = 0.043).

### 3.3. Analysis of the Causes of Death in AC Patients and the Pre- and Post-Transplant Complications

The leading causes of death and the mean age of the AC patients were also analyzed concerning the pre- and post-transplant complications (Table 4, Table S1).

The most frequent cause of death in AC patients was sepsis (21.3%) in younger patients (52.7 ± 1.5 years), while patients with a mean age of 54.0 ± 2.0 years presented with GFs as the more frequent cause (18.9%). Finally, multiorgan failure was observed in patients with a mean age of 59.2 ± 2.1 years as the more frequent cause, showing statistically significant differences between the distinct mean ages (*p* < 0.0001; Table 4).

Generally, the three main causes of death analyzed were more influenced by pre-transplant complications than by post-transplant complications. In the case of multiorgan failure, ascites were present in 77.8% and encephalopathy in 55.6% of cases, while only 26.3% had viral infections or acute rejection (AR; 26.3%). Similarly, when the deaths from sepsis were analyzed, it was observed that 72.7% of patients had ascites and 36.4% had encephalopathy. Finally, deaths due to GF mostly showed the presence of pre-transplant complications. Ascites were present in 92.3%, 42.9% of these patients showed the presence of encephalopathy (42.9%), and the presence of viral infections was 34.8%.

On the other hand, the mean ages among those who died due to multiorgan failure were also compared. We observed that the patients with ascites had a lower mean age of 56.1 ± 2.1 years compared to those with encephalopathy at 62.6 ± 4.3 years; these differences were statistically significant (*p* = 0.026: Table S1). Regarding the deaths from sepsis, statistically significant higher ages were observed among patients with ascites and encephalopathy compared to patients with viral infections (*p* = 0.034 and *p* = 0.016, respectively) or chronic rejection (*p* = 0.003 and *p* = 0.009). Similar results were found in deceased patients who died of GF.

**Figure 4:**
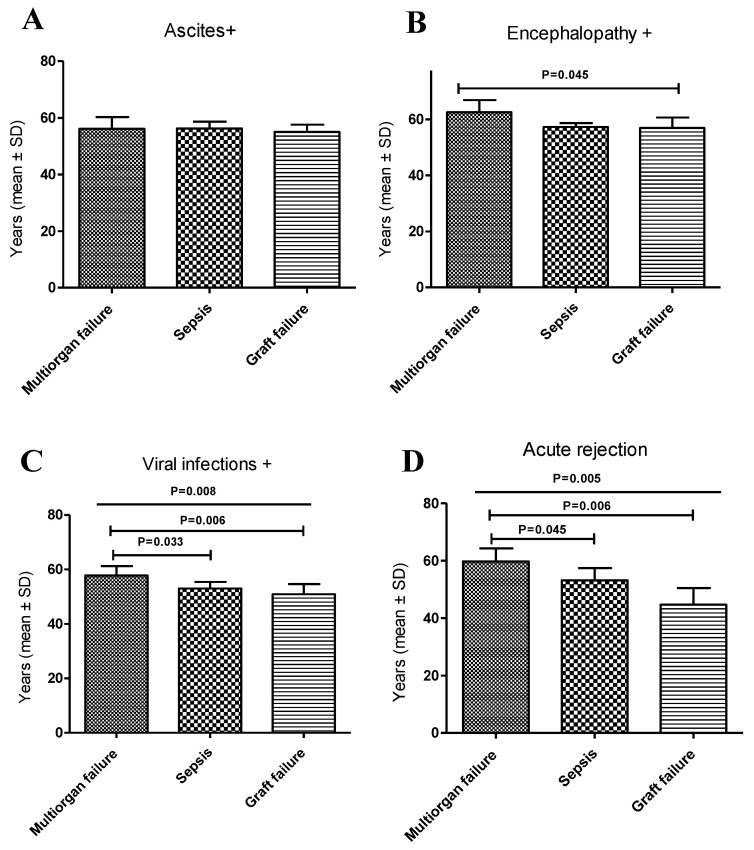
Analysis of the mean age of AC patients and its relationship with the main causes of death according to the different pre- and post-transplant complications. (**A**) Analysis of AC patients with ascites. (**B**) Analysis of AC patients with encephalopathy. (**C**) Analysis of AC patients with pre-transplant viral infections (HCV or HBV). (**D**)Analysis of AC patients with acute rejection.

The mean ages for the different types of deaths were also analyzed (Figure 4). In patients with ascites, the mean age was similar in the three types of deaths (Figure 4A). In AC patients with encephalopathy, an increase in the age of patients who died from multiorgan failure was observed compared to those who died from GF (*p* = 0.045; Figure 4B). In patients with viral infections, statistically significant differences were observed regarding the mean ages in the three types of death (*p* = 0.008; Figure 4C). The mean age of patients with multiorgan failure was higher than those who died from sepsis (*p* = 0.033) and GF (*p* = 0.006). Similar observations were obtained in the case of patients suffering acute graft rejection (*p* = 0.005). An increase in the mean age was observed in patients who died due to multiorgan failure compared to those who died due to sepsis (*p* = 0.045) and those who died due to GF (*p* = 0.006; Figure 4D).

Finally, the relationship between variables and survival for AC patients was analyzed using Cox regression multivariate analysis using the cause of death as a stratum. Significant *p*-values were found for ascites (*p* = 0.015) and chronic rejection (*p* = 0.037) (Table 5).

## 4. Discussion

Our study determined the different clinical and/or sociodemographic causes that influence the different causes of death and the short- and long-term survival of the AC patient undergoing liver transplantation. Our study results can help us to know a patient’s vulnerabilities in the hospital period after the liver transplant and outside of it. Close medical surveillance is essential in these patients for improving and adapting medical treatments to extend their survival in the short- and long-term and to avoid preventable deaths.

Most cirrhosis patients remain asymptomatic, their prognosis is reasonably good, and 5-year survival can reach up to 90% before decompensated cirrhosis is established [34,35]. Complications associated with portal hypertension, including ascites, spontaneous bacterial peritonitis (SBP), hepatic encephalopathy, hepatorenal syndrome, or pulmonary hypertension, have been documented in patients at this last stage [36,37,38]. In these cases, pre-transplant management’s main focus would be the complete elimination of the leading causes of cirrhosis, such as alcohol consumption [39].

Decompensated cirrhosis has a significant effect on prognosis with the subsequent development of ascites and hepatic encephalopathy, with a survival rate of 85% in the first year, 56% at five years, and just 50% at ten years [40,41]. In recent years, the survival rate of liver recipients suffering from AC has significantly improved, allowing this liver disease to be considered one of the best indications for LT. Patients should, however, undergo a comprehensive evaluation of post-transplant risk, taking into account the development of rejection, viral recurrence, or alcoholic relapse [18,42,43,44,45]. Forensic pathologists are familiar with substance addicts who have been found dead, and do not identify the cause of death [46]. In our study, the cases of registered deaths corresponded with non-sudden deaths (53.3%), followed by 27.8% of sudden deaths, and other unknown causes (18.9%).

Ascites are the product of portal hypertension, which induces the accumulation of water and sodium and the presence of excessive peritoneal fluid [36,47]. When complications arise, survival rates for patients with cirrhosis dramatically decrease, and for this reason, patients with decompensated cirrhosis are usually recommended for LT [48]. Our data showed that ascites were the complication with the highest frequency found, followed by encephalitis and viral infections; this observation was similar to that obtained by similar studies [1,49]. The AC patients with ascites did not show differences in terms of sex or age concerning the rest of the analyzed population, the latter being similar to other studies [24,25,29]. It should be noted that AC patients with ascites or encephalopathy and concomitant pre-transplantation viral infection had a lower mean age than AC patients with ascites and without viral infection. Therefore, it was observed that a viral condition accelerates an AC patient’s inclusion on the LT waiting list [50,51,52].

Our study observed that patients who developed post-transplant AR had a younger age than those without concomitant viral infections. This observation corroborates the data published by Cakaloglu, Y et al. [53] in 384 patients who developed AR with the presence of cytomegalovirus (CMV) and other viruses, including EBV, varicella-zoster virus, and hepatitis virus. In this study, all treated patients’ rejection process was managed with antiviral chemotherapy administered in rejection episodes with a concomitant viral infection.

On the other hand, the mean age of AC patients who died with concomitant viral infection due to non-sudden death shows a lower mean age than the rest of the deceased analyzed, this observation being also corroborated by other studies [53]. In our study, because the patients analyzed had been transplanted and subjected to retroviral treatments, their survival was more significant than in other studies where they analyzed the mortality burden of patients with chronic viral hepatitis, and their death certificates showed that they had died at an age greater than 20 years when compared with the deceased without HCV infection.

Our study shows that AC patients undergoing LT die from non-sudden deaths, the main cause being sepsis and GF. They also present less frequently with sudden death, with multiorgan failure being the primary cause. However, different studies highlight that alcohol abuse may contribute to a significant proportion of non-coronary sudden deaths [54,55] because ventricular arrhythmias are the most common mode of alcohol-related sudden death, including automaticity, triggering, and re-entry mechanisms. However, studies of the association between alcohol and sudden coronary death show that heavy drinking is linked to an increased risk of sudden death. Studies that do not take pre-existing ischemic heart disease into account are likely to underestimate the adverse effects of heavy drinking on the incidence of sudden death because the effects are not as evident [54]. Another study indicated that heavy drinkers tend to have higher total and cardiovascular mortality rates than light or moderate drinkers [56]. Therefore, as shown in our study, the benefit of liver graft transplantation on AC patients could translate into more prolonged survival and, perhaps due to alcoholic abstention and post-transplant treatments, death from cardiac arrest is no longer the leading cause of death, sepsis becoming the primary cause. Therefore, it would be necessary to carry out a protocol to avoid septic symptoms and GFs to avoid death and increase the AC patient’s survival.

There are complex and multifactorial mechanisms underlying the increased risk of infection and infection-related mortality in advanced liver disease, including compromised innate and adaptive immunity, bacterial overgrowth, dysbiosis, and the translocation of gut-resident bacteria and bacterial products [57,58]. Most immune cells’ functions can be modulated by alcohol use, which can undermine effective immune responses [59,60]. Innate immune system cells include neutrophils, monocytes, tissue-resident, recruited macrophages, dendritic cells, and natural killer (NK) cells.

Very few well-designed prospective trials have been tested according to the stage and clinical appearance of the microbiological characteristics of infection in alcoholic liver disease (ALD). However, this knowledge could help clinicians to better identify preventive and empirical antimicrobial measures, minimize the risk of infection, and increase the prognosis of patients with AC who are receiving LT [57]. Very few studies have also explicitly assessed the microbiological features of ALD infection according to the stage and clinical presentation. However, this information could help clinicians better define preventive and empirical antimicrobial strategies, decrease the risk of infection, and improve the prognosis of AC patients undergoing LT [57].

The analysis of ascites’ influence and the different causes of death show that AC patients without ascites showed early death from 6 months to 3 years of cardiac arrest and multiorgan failure.

Abnormalities in cardiac function have been reported in liver cirrhosis, suggesting latent cardiomyopathy in these patients [58,59]. Similar results were obtained in patients who suffered graft rejection, influencing patient mortality six months after transplantation. However, AC patients with encephalopathy did not show significant differences in survival according to the cause of death. However, a study analyzed the autopsy prevalence of Wernicke’s encephalopathy (WE) in alcohol-related disease, proving to be a frequent finding in people with alcohol-related diseases. They observed a high prevalence of WE in adult autopsies (6.6%) without documented clinical evidence that could have contributed to mortality in these cases [60].

One of the main limitations of the study, which could have been very interesting to analyze, was not having data on relapses in alcohol consumption in transplant patients. Furthermore, the registered data on mental health before and after transplantation in these patients were not available, so they could not be analyzed and taken into account. Despite all these limitations, statistically robust analyses with promising diagnostic implications have been achieved in this study.

In conclusion, our results show that a large proportion of the deaths of AC patients undergoing transplantation is due to non-sudden death, sepsis, and GF, the main causes of the sample being similar in both sexes. Multiorgan failure is the main cause of sudden death, and the one with the highest percentage of post-transplant mortality during the first years after transplantation, followed by sepsis and GF. In non-sudden deaths, there are no significant differences between the death rates related or not to the transplanted liver. Furthermore, our results show how pre-transplant clinical complications, such as viral infections and encephalopathy, influence the age at which multiorgan failure occurs in the transplanted patient.

In conclusion, our results show AC patient’s vulnerability not only in the hospital period after the transplant but also outside of it. Close medical surveillance and the search for biomarkers that allow for predicting susceptibility to a specific cause of death are essential in these types of patients to improve and adapt medical treatments to extend their survival, both in the short- and long-term.

## Figures and Tables

**Table 1:** Baseline demographic, clinical, and biochemical characteristics of the male AC patients were analyzed in this study.

	Total AC Patients *N* = 122, *n* (%)
Mean age (mean years ± SD)	53.1 ± 8.9
Sex (male/female)	113 (92.6)/9 (7.4)
Pre-transplantation complications	
Ascites +, *n* = 41 Mean age *	41/50 (82.0) 54.2 ± 1.3
Grade	
I	8 (19.5)
II	18 (43.9)
III	15 (36.6)
Encephalopathy +, *n* = 21 Mean age *	21/52 (40.4) 56.7 ± 2.0
Grade	
I	9 (42.8)
II	10 (47.7)
III	2 (9.5)
Viral infection ^a^ +, *n* = 122 Mean age *	33/122 (27.0); 51.3 ± 1.9
Biochemical parameters	
Creatinine (mg/dL)	1.12 ± 0.35
Albumin (g/dL)	3.23 ± 0.07
Total bilirubin (mg/dL)	3.01 ± 0.19
GOT (U/L)	97.23 ± 12.67
GPT (U/L)	70.8 ± 9.56
GGT (U/L)	98.96 ± 7.10
AP (U/L)	172.09 ± 8.32
Prothrombin activity (%)	59.08 ± 0.97
INR	1.47 ± 0.22
MELD	14.4 ± 0.7
Child–Pugh % (A/B/C)	18.4/52.4/29.5

AC, alcoholic cirrhosis; AP, alkaline phosphatase; GGT, γ-glutamyl transferase; GOT, glutamic oxaloacetic transaminase; GPT, glutamic pyruvic transaminase; INR, international normalized ratio; MELD, model for end-stage liver disease; N, total number of individuals; n, number of patients in each group; SD, standard deviation. * The mean values were analyzed (mean value ± SD) in all cases; ^a^ patients with viral infection, including HCV and HBV. No grade IV patients with encephalopathy were found in any case.

**Table 2:** Causes and time of death of AC patients undergoing liver transplantation by sex and viral infection pre-transplant.

		AC Patients by Sex				AC Patients by Viral Infection		
	Total AC Patients * N = 122, *n* (%) Mean ± SD	Men *N* = 113, *n* (%) Mean ± SD	Women *N* = 9, *n* (%) Mean ± SD	*p* **	OR (95% CI)	Virus *** N = 33, *n* (%) Mean ± SD	Non-virus *N* = 89, *n* (%) Mean ± SD	*p* **
Mean age (years)	53.1 ± 8.9	53.2 ± 9.1	50.7 ± 6.2	0.274	-	54.2 ± 0.9	49.9 ± 1.6	**0.019**
Pre-transplant complications								
Ascites +	41/50 (82.0) ^a^ 54.2 ± 1.3	38/47 (80.9) ^b^ 54.6 ± 1.4	3/3 (100) 50.0 ± 4.6	1.000 0.369	1.079 (0.990–1.176) -	15/41 (36.6) ^c^ 50.4 ± 2.0	26/41 (63.4) ^d^ 56.4 ± 1.6	0.715 **0.024**
Encephalopathy +	21/52 (40.4) 56.7 ± 2.0	21/49 (42.9) 57.0 ± 1.9	0/3 (0) -	0.264 -	0.903 (0.805–1.014) -	5/21 (23.8) 47.2 ± 2.8	16/21 (76.2) 60.1 ± 1.8	0.149 **0.002**
Viral infection +	33/122 (27.0) 51.3 ± 1.9	30/113 (26.5) 50.0 ± 1.7	3/9 (33.3) 49.0 ± 6.3	0.702 0.854	1.383 (0.325–5.882) -	- -	- -	- -
Post-transplant complications								
Acute rejection	25/122 (20.5) 54.3 ± 3.0	23/113 (20.4) 51.3 ± 2.1	2/9 (22.2) 54.5 ± 0.5	1.000 0.664	1.118 (0.218–5.745) -	8/25 (32.0) 45.4 ± 3.6	17/25 (68.0) ^e^ 54.4 ± 2.1	0.615 **0.029**
Chronic rejection	13/122 (10.7) 52.8 ± 2.4	13/113 (11.5) 50.7 ± 2.3	0/9 (0) -	0.595 -	0.917 (0.867–0.971) -	0/13 (0) -	13/13 (100) 50.7 ± 2.3	**0.019** -
Cause of death								
Sudden death	34 (27.9) 55.2 ± 9.9	31 (27.4) 56.3 ± 9.6	3 (33.3) 44.7 ± 6.1	0.708 0.051	0.756 (0.178–3.211) -	8/34 (23.5) ^f^ 55.5 ± 3.5	26/34 (76.5) ^g^ 55.2 ± 2.0	0.827 0.933
Non-sudden death	65 (53.2) 51.9 ± 9.0	60 (53.1) 51.8 ± 9.3	5 (55.5) 53.4 ± 4.2	1.000 0.500	0.906 (0.231–3.549) -	19/65 (29.2) 47.3 ± 2.0	46/65 (70.8) 53.9 ± 1.3	0.827 **0.007**
Unknown	23 (18.9) 52.9 ± 6.8	22 (19.5) 52.9 ± 7.0	1 (11.1) 55.0 ± 0.0	1.000 0.768	1.934 (0.230–16.281) -	6/23 (26.1) 50.8 ± 2.8	17/23 (73.9) 53.7 ± 1.7	0.827 0.389
Time of death								
≤1 month	14 (11.8) ^h^ 53.1 ± 10.8	13 (11.7) ^i^ 52.6 ± 11.0	1 (12.5) ^j^ 60.0 ± 0.0	0.305 0.531	0.455 (0.085–2.428) -	5/14 (35.7) ^k^ 56.8 ± 3.0	9/14 (64.3) ^l^ 51.1 ± 4.1	0.697 0.364
˃1–6 months	44 (37.0) 55.7 ± 9.3	39 (35.1) 56.3 ± 9.8	5 (62.2) 51.4 ± 2.1	0.719 0.012	0.659 (0.167–2.595) -	12/44 (27.3) 49.1 ± 2.6	32/44 (72.7) 58.2 ± 1.5	0.697 0.003
≥6 months–10 years	61 (51.3) 51.4 ± 7.8	59 (53.2) 51.7 ± 7.7	2 (25.0) 42.0 ± 5.7	0.306 0.082	2.346 (0.559–9.847) -	15/61 (24.6) 49.4 ± 2.2	46/61 (75.4) 52.0 ± 1.1	0.697 0.259

AC, alcoholic cirrhosis; N, total number of individuals; n, number patients in each group. Comparisons were made by the two-sided Fisher’s exact test and two-sided Student *t* test. * Total individuals who died at 10 years post-transplant. ** the *p*-value shown in this table was obtained by comparing male and female patients. A level of *p* < 0.05 was accepted as statistically significant. OR, odds ratio with a confidence interval (CI) of 95%. *** HCV and HCB were included. Comparisons were made between the mean age of the presence of ascites, encephalopathy, and viral infections ^a^ *p* < 0.000; ^b^ *p* < 0.000; ^c^ *p* = 0.012; ^d^ *p* < 0.000. Comparisons were made between the mean age of non-virus patients with acute and chronic rejection; ^e^ *p* = 0.013. Comparisons were made between the different causes of death in viral AC patients; ^f^ *p* = 0.000. Comparisons were made between the different causes of death in non-viral AC patients; ^g^ *p* = 0.002. Comparisons were made between the mean ages at different times of death (≤1 month, ≥ 1–6 months, ˃6 months–10 year) in total and by sex; ^h^ *p* = 0.049; ^i^ *p* = 0.044; ^j^ *p* = 0.013. Comparisons were made between the mean ages at different times of death (≤1 month, ≥1–6 months, ˃6 months–10 year) in viral AC patients (^k^ *p* = 0.000) and non-viral AC patients (^l^ *p* = 0.000).

**Table 3:** Causes and time of death in AC patients undergoing liver transplantation in the short- and long-term.

	Time of Death								
	≤One Month	≥1–6 Months	6 Months	1 Year	3 Years	5 Years	10 Years		
Cause of Death, n (%)	*N* = 14, *n* (%)	*N* = 43, *n* (%)	*N* = 57, *n* (%)	*N* = 74, *n* (%)	*N* = 99, *n* (%)	*N* = 110, *n* (%)	Total * N = 122, *n* (%)	Men *N* = 113, *n* (%)	Women *N* = 9, *n* (%)
Sudden death	2 (14.3)	15 (34.9)	17 (29.8)	21 (28.4)	26 (26.3)	33 (30.0)	34 (27.8)	31 (27.4)	3 (33.3)
Non-liver related	2 (14.3)	15 (34.9)	17 (29.8)	21 (28.4)	26 (26.3)	33 (30.0)	34 (27.8)	31 (27.4)	3 (33.3)
Cardiac arrest	-	1 (6.7)	5 (29.4)	2 (9.5)	3 (11.5)	6 (18.2)	6 (17.6)	4 (13.0)	2 (66.7)
Digestive bleeding	-	2 (13.3)	5 (29.4)	2 (9.5)	5 (19.3)	6 (18.2)	7 (20.5)	7 (22.5)	-
Edema lung	-	-	2 (11.8)	-	1 (3.8)	2 (6.1)	2 (5.9)	2 (6.5)	-
Multiorgan failure	2 (100)	12 (80.0)	5 (29.4)	17 (81.0)	17 (65.4)	19 (57.5)	19 (56.0)	18 (58)	1(33.3)
Non-sudden death	10 (71.4)	24 (55.8)	34 (59.7)	41 (55.4)	54 (54.5)	57 (51.8)	65 (53.3)	60 (53.1)	5 (55.6)
Non-liver related	5 (50.0)	15 (30.2)	17 (31.6)	21 (28.4)	31(31.3)	33 (30.0)	37 (30.4)	33 (29.1)	4 (44.5)
Metastasis	-	1 (6.7)	5 (29.4)	1 (4.7)	4 (13.0)	5 (15.2)	6 (16.2)	6 (18.1)	-
Neoplasia pharynx	-	-	1 (5.9)	-	1 (3.2)	1 (3.0)	1 (2.7)	1 (3.0)	-
Pancreatitis	-	1 (6.7)	-	1 (4.7)	1 (3.2)	1 (3.0)	1 (2.7)	1 (3.0)	-
Pneumonia	-	1 (6.7)	2 (1.8)	2 (9.6)	3 (9.6)	3 (9.1)	3 (8.1)	3 (9.1)	-
Sepsis	5 (50.0)	12 (80.0)	9 (52.9)	17 (81.0)	22 (71.0)	23 (69.7)	26 (70.3)	22 (66.8)	4 (100)
Liver related	5 (35.7)	10 (25.6)	16 (28.1)	20 (27.0)	23 (23.2)	24 (21.8)	28 (22.9)	27 (24.0)	1 (11.1)
Chronic rejection	-	-	2 (16.7)	-	-	1 (4.2)	2 (7.1)	2 (7.4)	-
Graft failure	4 (80.0)	9 (90.0)	9 (75.0)	18 (90.0)	21 (91.4)	21 (87.4)	23 (82.0)	22 (81.5)	1 (100)
Primary dysfunction	1 (20.0)	-	1 (8.3)	1 (5.0)	1 (4.3)	1 (4.2)	2 (7.1)	2 (7.4)	-
Viral relapse (HCV)	-	1 (10.0)	-	1 (5.0)	1 (4.3)	1 (4.2)	1 (3.8)	1 (3.7)	-
Unknown	2 (14.3)	4 (9.3)	15 (10.5)	12 (16.2)	19 (19.2)	20 (18.2)	23 (18.9)	22 (19.5)	1 (11.1)

AC, alcoholic cirrhosis; N, total number of individuals dead at 10 years; n, number of patients in each group; HCV, hepatitis C virus. * Total accumulated deaths.

**Table 4:** Main causes of death in AC patients and its relationship with pre- and post-transplant complications.

		Pre-Transplant Complications			Post-Transplant Complications	
Main Causes Of Death N (%), (Mean Years ± SD)	Total ^a^	Ascites +	Encephalopathy +	Viral Infections + ^b^	AR	CR
Multiorgan failure, n (%)	19 (15.6) 59.2 ± 2.1 ^c^	7/9 (77.8) 56.1 ± 4.2	5/9 (55.6) 62.6 ± 4.3 ^d^	5/19 (26.3) 57.8 ± 3.4 ^e^	5/19 (26.3) 59.8 ± 4.5 ^f^	0/19 (0) -
Sepsis, n (%)	26 (21.3) 52.7 ± 1.5	8/11 (72.7) 56.3 ± 2.4	4/11 (36.4) 57.3 ± 1.4	5/26 (19.2) 53.0 ± 2.4	5/26 (19.2) 53.2 ± 4.3	4/26 (15.4) 49.0 ± 4.1
Graft failure, n (%)	23 (18.9) 54.0 ± 2.0	12/13 (92.3) 55.0 ± 2.6	6/14 (42.9) 57.0 ± 3.7	8/23(34.8) 50.9 ± 3.7	3/23 (13.0) 44.7 ± 5.8	2/23 (8.7) 45.5 ± 4.5

Ascites +, presence of ascites; Encephalopathy +, presence of encephalopathy; AC, alcoholic cirrhosis; AR, acute rejection; CR, chronic rejection; HCV, hepatitis C virus; HBV, hepatitis B virus; N, total number of individuals; n, number of patients in each group. Comparisons were made by a two-sided Fisher’s exact test, two-sided Student *t* test, and an ANOVA test. Sums of squares (SS), degrees of freedom (df), mean squares (MS), and F and *p*-values were obtained by comparing the main causes of death. A level of *p* < 0.05 was accepted as statistically significant. ^a^ Total individuals who died at 10 years’ post-transplant. ^b^ HCV and HCB were included in viral Infections group. ^c^ Comparing the mean age of the total causes of deaths: *p* < 0.000, F: 72.021, MS:247.784, df: 2, SS: 495.568. ^d^ Comparing the mean age of the total causes of deaths in presence of encephalopathy: *p* = 0.045, F: 4.059, MS:50.159, df: 3, SS:100.317 ^e^ Comparing the mean age of the total causes of deaths in AC patients with pre-transplant viral infections: *p* = 0.008, F: 6.705, MS: 73.800, df: 2, SS: 147.600 ^f^ Comparing the mean age of the total causes of deaths in AC patients with AR: *p* = 0.005, F: 9.679, MS: 215.112, df: 2, SS:430.223.

**Table 5:** Cox regression multivariate analysis for AC patient’s survival.

	Wald	*p*	OR	95% CI	
				Lower	Upper
Age	0.150	0.698	1.010	0.961	1.061
Sex	1.610	0.204	2.686	0.584	12.354
Ascites	5.880	0.015	4.487	1.334	15.099
Encephalopathy	2.632	0.105	2.007	0.865	4.658
Viral infection (HCV/HBV)	0.151	0.698	1.206	0.468	3.105
Acute rejection	1.368	0.242	1.822	0.667	4.977
Chronic rejection	4.329	0.037	0.111	0.014	0.880

OR, odds ratio with a confidence interval (CI) of 95%. *p*-values marked in bold are statistically significant at a level of *p* ˂ 0.05.

## Data Availability

Not applicable.

## Supplementary Materials

The following are available online at https://www.mdpi.com/article/10.3390/diagnostics11060968/s1, Table S1: Comparison of mean ages between main causes of death in AC patients and pre- and post-transplant complications.
