# Supplementary material for: Causes of Death and Survival in Alcoholic Cirrhosis Patients Undergoing Liver Transplantation: Influence of the Patient’s Clinical Variables and Transplant Outcome Complications
Source: Diagnostics (Basel). 2021 May 27;11(6):968. doi: 10.3390/diagnostics11060968 (PMC8227029; doi:10.3390/diagnostics11060968)
Supplement: Supplementary file 1 [file diagnostics-11-00968-s001.zip › diagnostics-1207874-supplementary.pdf]

Supplementary Table S1. Comparison of mean ages between main causes of death in AC patients and pre- and post-transplant complications.

	P	SS	df	MS	F
**Multiorgan failure**					
Ascites+ *vs* encephalopathy+	**0.026**	123.229	1	123.229	6.854
Ascites + *vs* viral infections[a]	0.474	8.429	1	8.429	0.554
Encephalopathy+ *vs* viral infections	0.086	57.600	1	57.600	3.834
AR *vs* ascites+	0.174	39.929	1	39.929	2.137
AR *vs* encephalopathy+	0.344	19.600	1	19.600	1.012
AR *vs* viral infections	0.451	10.000	1	10.000	0.629
**Sepsis**					
Ascites+ *vs* encephalopathy+	0.465	2.667	1	2.667	0.577
Ascites+ *vs* viral infections	**0.034**	33.508	1	33.508	5.817
Encephalopathy+ *vs* viral infections	**0.016**	41.089	1	41.089	9.945
AR *vs* ascites+	0.120	29.569	1	29.569	2.846
AR *vs* encephalopathy+	0.113	37.356	1	37.356	3.275
AR *vs* viral infections	0.930	0.100	1	0.100	0.008
AR *vs* CR	0.181	39.200	1	39.200	2.206
CR *vs* ascites+	**0.003**	142.107	1	142.107	15.659
CR vs encephalopathy+	**0.009**	137.780	1	137.780	14.681
CR *vs* viral infections	0.108	35.556	1	35.556	3.388
**Graft failure**					
Ascites+ *vs* encephalopathy+	0.199	16.000	1	16.000	1.793
Ascites + *vs* viral infections	**0.009**	80.688	1	80.688	8.534
Encephalopathy+ *vs* viral infections	**0.010**	127.577	1	127.577	9.319
AR *vs* ascites+	**0.000**	254.616	1	254.616	23.369
AR *vs* encephalopathy+	**0.006**	302.580	1	302.580	15.605
AR *vs* viral infections	0.060	83.869	1	83.869	4.628
AR *vs* CR	0.881	0.768	1	0.768	0.026
CR *vs* ascites+	**0.001**	154.714	1	154.714	19.623
CR *vs* encephalopathy+	**0.011**	198.375	1	198.375	13.419
CR *vs* viral infections	0.111	46.656	1	46.656	3.215

Ascites+, Presence of ascites; Encephalopathy+, Presence of encephalopathy; AC, alcoholic cirrhosis; AR, acute rejection, CR; Chronic rejection; HCV; Hepatitis C Virus; HBV; Hepatitis C Virus; N, total number of individual; n, number patients in each group. Comparisons were made by ANOVA test. Sums of squares (SS), degrees of freedom (df), mean squares (MS), and F and P-values was obtained by comparing the main causes of death in AC patients and pre- and post-transplant complications. A level of $P< 0.05$ was accepted as statistically significant. [a]HCV and HCB were included in viral Infections group.
